# Integrity of dural closure after autologous platelet rich fibrin augmentation: an in vitro study

**DOI:** 10.1007/s00701-020-04254-4

**Published:** 2020-02-07

**Authors:** I. Vasilikos, J. Beck, S. Ghanaati, J. Grauvogel, T. Nisyrios, K. Grapatsas, U. Hubbe

**Affiliations:** 1 Department of Neurosurgery, Medical Centre-University of Freiburg, Faculty of Medicine, University of Freiburg, Neurozentrum, Breisacher Str. 64, D-79106 Freiburg, Germany; 2 Laboratory of Experimental Neurosurgery, Medical Centre-University of Freiburg, Faculty of Medicine, University of Freiburg, Neurozentrum, Breisacherstr. 64, Freiburg, Germany; 3 Frankfurt Oral Regenerative Medicine, Clinic for Maxillofacial and Plastic Surgery, Johann Wolfgang Goethe University, Frankfurt am Main, Germany; 4 Department of Oral and Craniomaxillofacial Surgery, University Medical Centre Freiburg, Freiburg, Germany; 5 Department of Thoracic Surgery, Faculty of Medicine, Medical Centre-University of Freiburg, Freiburg, Germany

**Keywords:** Watertight Dural closure, Dural onlays, Autologous biomaterial, Neurosurgery

## Abstract

**Background:**

Watertight closure of the dura mater is fundamental in neurosurgery. Besides the classical suturing techniques, a variety of biomaterials have been proposed as sealants. Platelet rich fibrin (PRF) is an autologous biomaterial which can readily be obtained through low-speed centrifugation of patient’s own blood. It is rich in fibrin, growth factors, leucocytes and cytokines and has shown adhesive properties while promoting the physiological wound healing process. In this study, we investigated the effect of applying PRF in reinforcing the watertight dura mater closure.

**Methods:**

We created an in vitro testing device, where the watertight dura mater closure could be hydrostatically assessed. On 26 fresh harvested bovine dura maters, a standardised 20-mm incision was closed with a running suture, and the leak pressure was measured first without (primary leak pressure) and then with PRF augmentation (secondary leak pressure). The two groups of measurements have been statistically analysed with the Student’s paired *t* test.

**Results:**

The “running suture only group” had a leak pressure of 10.5 ± 1.2 cmH2O (mean ± SD) while the “PRF-augmented group” had a leak pressure of 47.2 ± 2.6 cm H2O. This difference was statistically significant (*p* < 0.001; paired *t* test).

**Conclusions:**

Autologous platelet rich fibrin augmentation reliably reinforced watertight closure of the dura mater to a > 4-fold increased leak pressure after failure of the initial standard running suture technique.

**Electronic supplementary material:**

The online version of this article (10.1007/s00701-020-04254-4) contains supplementary material, which is available to authorized users.

## Introduction

To avoid complications associated with cerebrospinal fluid (CSF) leak after neurosurgical operations, watertight dura mater closure is fundamental [1]. Postoperative CSF leakage can result in meningitis, wound-related problems and other complications such as intracranial hypotension. In addition, dural healing is furthermore important for future revision-operations or possible adjuvant treatments in the operated area. Various suturing techniques and reinforcements with bio-products have been suggested and tested over the years [13, 12, 18–20].

Platelet rich fibrin (PRF) is a new generation of autologous platelet concentrates, without biochemical blood handling. It is rich in fibrin, leukocytes, cytokines and growth factors which accelerate and promote the natural wound healing while preventing the local occurrence of infections [3, 7, 9–11, 18, 19, 31]. First generation of blood concentrates systems such as plasma-rich growth factors (PRGF) and platelet-rich plasma (PRP) involved secondary processing with non-autologous anticoagulants, multi-step centrifugations and elimination of leucocytes [2, 14, 30].

PRF serves as an autologous bio-scaffold and reservoir of growth factors for tissue regeneration, allowing cells to migrate and proliferate faster [22]. All the known clinical applications of PRF highlight an accelerated tissue healing due to the development of effective neovascularisation, accelerated wound closing with fast cicatricial tissue remodelling and reduction of infectious events [6].

PRF can be prepared in two different textures either fluid or solid, according to the updated protocol of Choukroun and Ghanaati in 2018, after a single cycle of low-speed centrifugation [5].

**Fig. 1:**
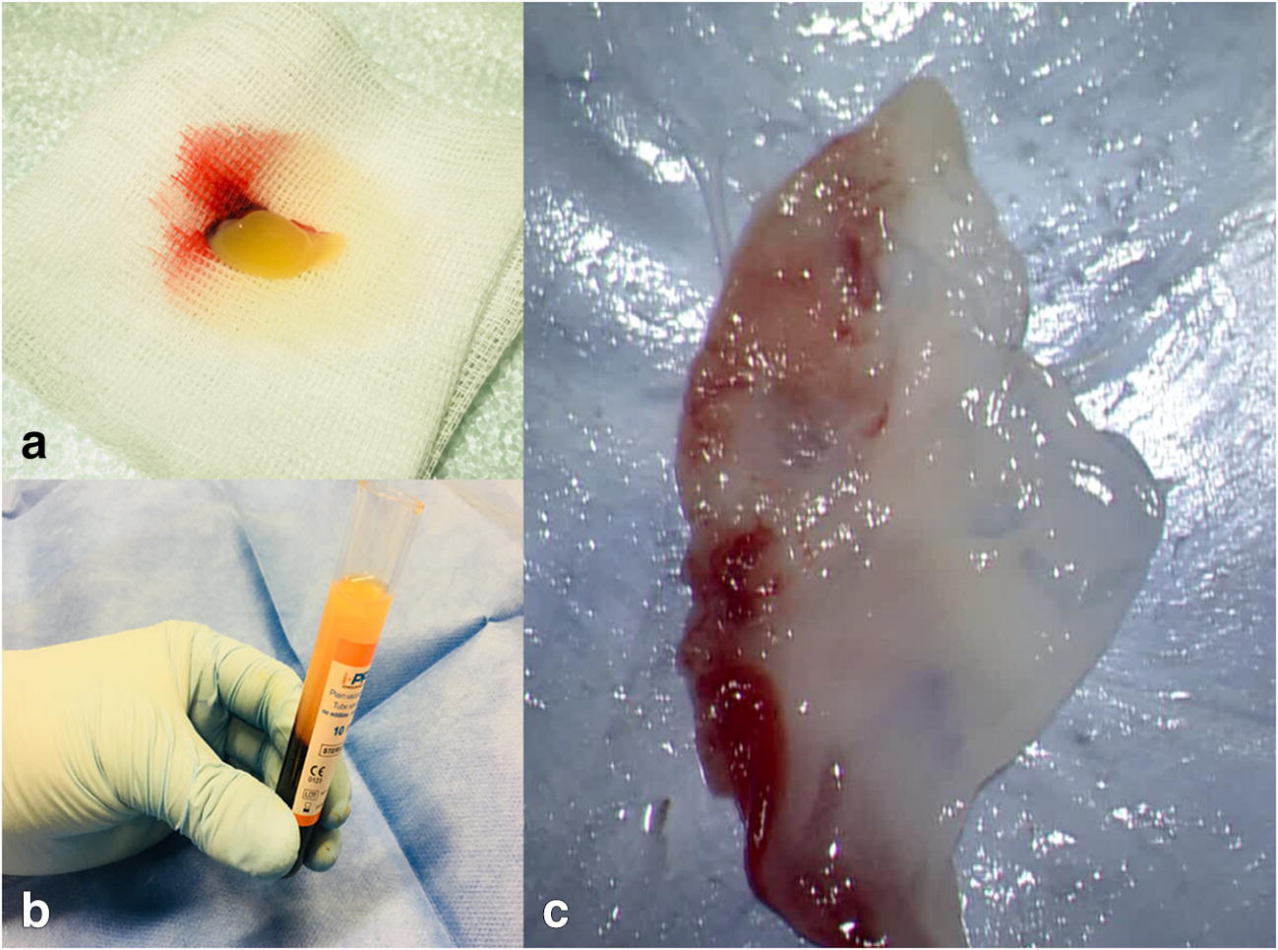
(A) s-PRF resulting fibrin clot, (B) Isolation of the i-PRF. (C) s-PRF fibrin clot after flattening through mechanical compression

The solid-PRF (s-PRF, Fig. 1(A, C) comprises an elastic 3D fibrin-matrix. It can be shaped through mechanical manipulation as needed, to fit its use. The injectable-PRF (i-PRF, Fig. 1(B)) comprises a fibrin matrix in an intermediate phase. i-PRF has a high viscosity and gradually becomes gelatinous after removal from its preparation vial.

In addition to its wound-healing properties, autologous fibrin is widely used in various surgical fields (ophthalmology, orthopaedic and vascular surgery) as a sealant due to its tissue-adhesive properties. Consequently, PRF may serve as an intraoperative glue to immediately enhance the watertightness of dural suture [8, 15, 17, 32].

In this study, we present an in vitro set-up to investigate, whether augmenting the classical dura suture with a combination of PRF in both states can result in a stronger watertightness in cases of simulated increased intracranial pressures.

## Materials and methods

### Dura testing device and sample installation

**Fig. 2:**
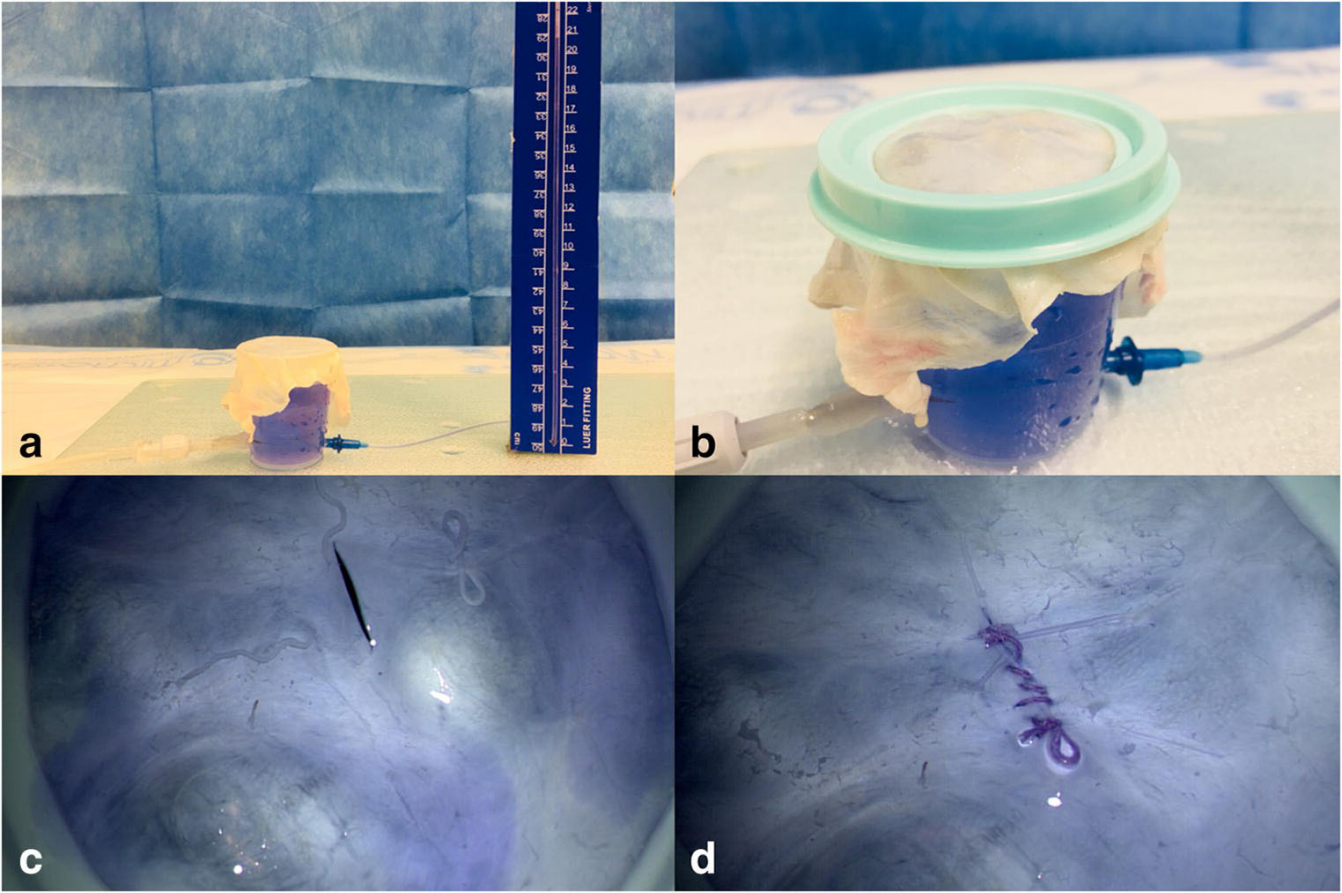
(A) Application of the dural sample on the testing chamber. (B) Clammed dural sample. (C) 20-mm dural incision and air decompression by filling up the chamber. (D) Dural sample after running suture

We developed a testing device to determine the leak pressure of a dural suture. (Fig. 2(A, B)).

A transparent plastic reversed flat top cone-shaped container with an opening of 4.5 cm was equipped with two luer lock connections, both ~ 1 cm above the bottom. One was used as input through which Evans-blue-dyed saline solution could be injected with a syringe. The other was connected to a hydrostatic pressure measuring column to assess the intra-chamber pressure. The collected dura samples were placed as shown in Fig. 2(A) on the opening of the container and then water-tight clamped with a tightly fitting plastic ring Fig. 2(B).

Twenty-six fresh bovine dura preparations (10 cm × 10 cm) were harvested from 4- to 6-month-old bovine skulls, from the central meat processing unit in the city of Freiburg and kept refrigerated at 4 °C in saline. All experiments were conducted within 1 week after the dural harvesting.

### Preparation of solid and injectable PRF biomaterial

For every dura closure, we used one vial for solid and one for injectable PRF, each containing 10 ml of healthy volunteer’s human blood and centrifuged in the DUO centrifuge (process for PRF, Nice, France) according to the low centrifugation concept protocol of Choukroun and Ghanaati [5]. This centrifuge has a fixed angle rotor with a radius of 110 mm. According to the aforementioned protocol, blood was centrifuged at 1200 rpm for 8 min (177 g). All donors had no signs of infectious disease. None of the healthy volunteers used any anticoagulants.

Sterile plastic tubes (process for PRF, Nice, France), with a volume of 10 ml were used for the preparation of the fluid (injectable) PRF, while 10-ml sterile glass tubes (process for PRF, Nice, France), were used for the preparation of the solid PRF. Both tubes were centrifuged as previously described for 8 min at 1200 rpm. Blood was drawn by means of a clinically approved butterfly blood collection method. Centrifugation was initiated with no delays, directly after blood collection, over a total time of 2–3 min maximum.

After completion of the centrifugation process, tubes were left to rest for 5 min in the device. From the glass tubes, a solid PRF matrix (s-PRF) was collected (Fig. 1(A)) and then manually compressed and flattened (Fig. 1(C)). From the plastic tubes, a thick fluid version of the injectable PRF (i-PRF), was aspired in a 10-ml syringe [5].

### Measurement of fluid leak pressure

After fixation of the dura samples to the testing device, a validation of the watertight fixation and sample-integrity was performed. No water leak was observed after reaching a pressure of up to 70 cm H2O.

A straight 20-mm incision was performed in the centre of the dura with a number 11 scalpel. The container was then filled to the rim with the blue-dyed saline until no residual air was trapped in the system (Fig. 2(C)).

The incision was sutured with Premicron 4–0 running simple closure suture (with 2 to 3 throws per centimetre using 3 to 4 mm bites, Fig. 2(D)) according to the standards for dura-closure during neurosurgical operations (University Clinic of Freiburg, Department of Neurosurgery). The specification including the quantity of stitches was the same for all sample closures.

After completion of suturing the incised dura-sample, pressure in the chamber was slowly increased by injection of more dyed-saline through the input valve. At the same time the hydrostatic pressure column measured, the intra-chamber pressure increase. By the first visual confirmation of a dura leak through the sutured dura, the measured intra-chamber pressure was documented and defined as the primary-leak-pressure. Then the pressure was again slowly decreased to the point that no dyed water remained above the sutured dura.

**Fig. 3:**
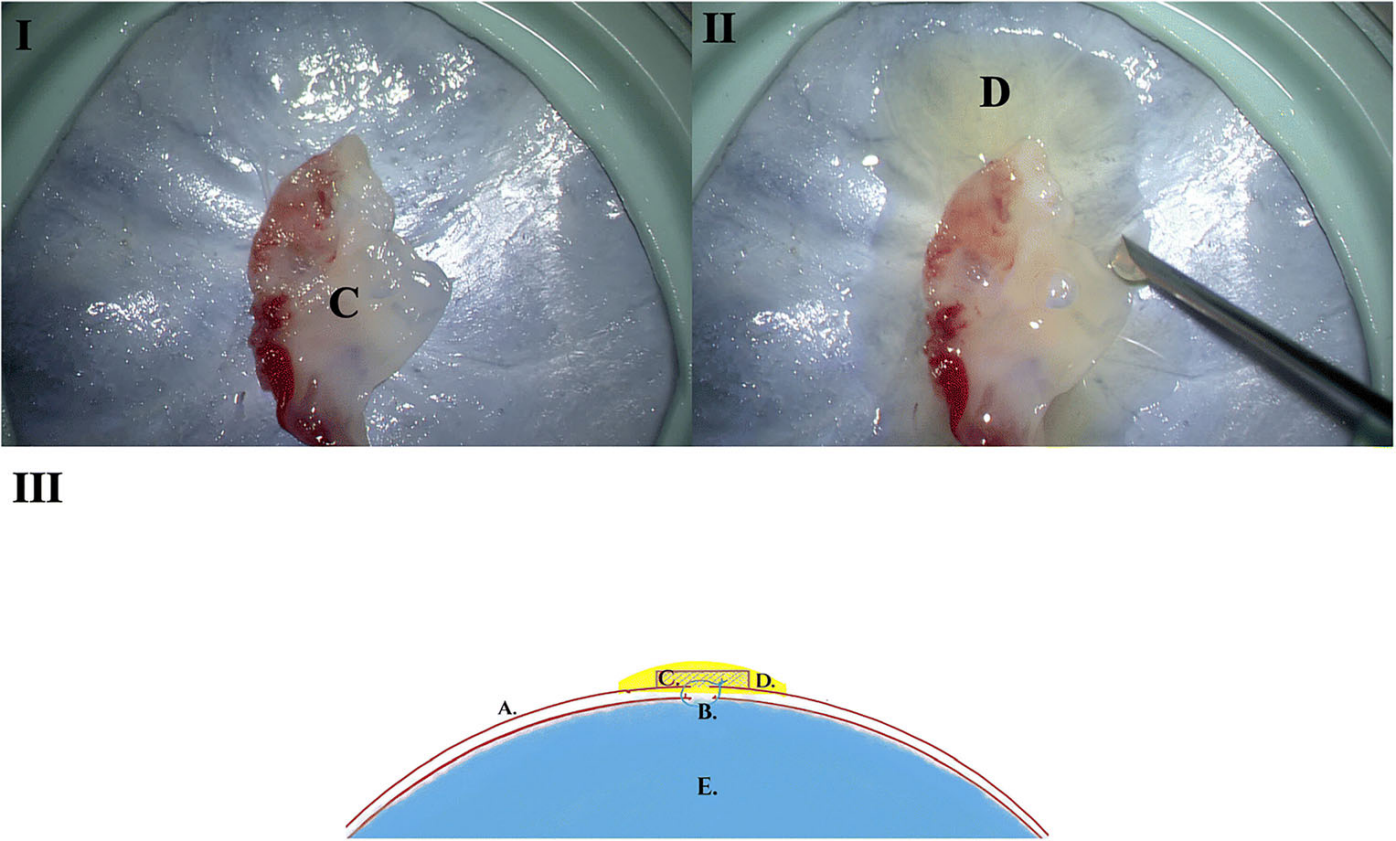
(I) s-PRF membrane applied on the sutured area. (II) Application of the i-PRF on top of the s-PRF membrane. (III) Schematic 1: (A) Dura mater. (B) Dura-suture. (C) s-PRF. (D) i-PRF. (E) Cerebrospinal fluid

In the second phase of the experiment, the flattened solid PRF membrane was applied on top of the sutured incision covering it completely as shown in Fig. 3(I, III).

Immediately after that, the previously collected injectable PRF was applied drop by drop slowly on top creating a thin layer over the solid PRF membrane (Online Resource - video 1). After 1 min, we proceeded with the same algorithm of gradually increasing the intra-chamber pressure. By the first visual confirmation of a dura leak through the sutured dura, the measured intra-chamber pressure was documented and defined as the secondary-leak-pressure. (Fig. 3(II) and Online Resource - video 2). During this phase of the experiment, two observers were monitoring the chamber and the pressure column respectively. In order to avoid internal errors in cases where small leaks could be masked through the PRF onlay, situations of pressure drop on the column without simultaneous observed leak on the chamber were documented as leak.


ESM 1(MPG 68944 kb)

The whole process was visualised and videodocumented with a neurosurgery microscope (Pentero 900 from Zeiss Inc., Superlux® 330 light source with 2 × 300 W xenon). The distance between the microscope and dura mater was 10 cm.

### Statistical analysis

Comparison of primary and secondary leak pressure of suture only and PRF-augmented closure was performed using Student’s paired *t* test. A *p* value < 0.05 was considered significant.

## Results

**Fig. 4:**
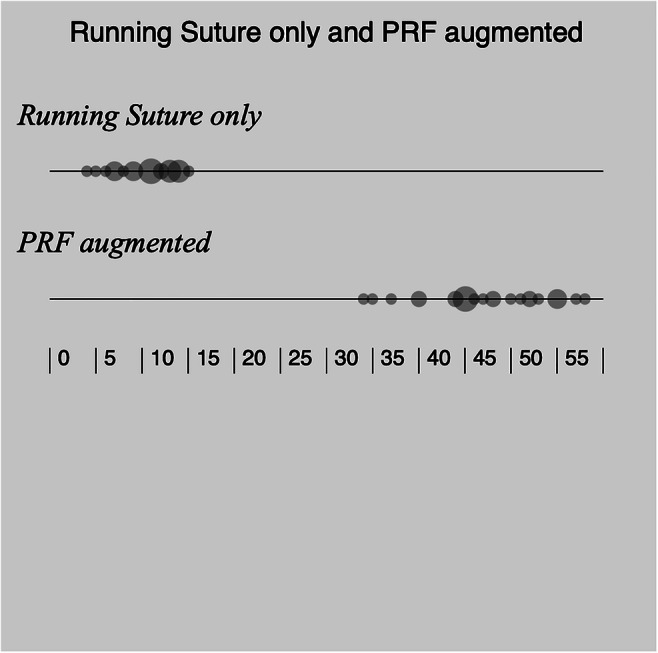
Dot plot illustrating leak-pressure measurements (cm H2O) for both closure methods

Primary and secondary leak pressures are presented in Table 1. The “running suture only group” had a mean pressure of 10.5 ± 1.2 cmH2O while the “PRF-augmented group” had a significantly higher mean value of 47.1 ± 2.6 cm H2O (*p* < 0.001; paired *t* test) (Fig. 4). In all measurements, the visualised leak on the chamber from the first observer was verified with a concomitant pressure drop on the column recorded by the second observer. In all cases, liquid PRF fully covered the surface of the chamber and the underlying PRF membrane. The concentration of Fibrin on each PRF-application was not evaluated during this study, neither the serum-Fibrin levels of each volunteer. Due to the masking effect of the PRF onlay, a precise localisation of the leak origin along the sutured defect was not possible. In many situations, a small leak was observed under the liquid PRF onlay without bursting through and disrupting its continuity. In these specimens (2, 15 and 16), the initially recorded pressure drop was documented as secondary leak pressure.

**Table 1:** Primary and secondary leak pressure measurements

	Pressure in cmH2O before fluid leak	
Specimen	Dura-closure with running suture only Primary leak pressure	PRF augmented dura-closure Secondary leak pressure
1	12	40
2	8	35
3	11	45
4	13	55
5	9	52
6	14	55
7	12	45
8	11	48
9	7	47
10	4	40
11	15	55
12	14	45
13	13	50
14	11	44
15	6	37
16	9	34
17	7	44
18	9	48
19	14	52
20	11	45
21	13	46
22	5	45
23	14	51
24	11	53
25	13	58
26	7	57

## Discussion

We could show that autologous platelet rich fibrin (PRF) reliably reinforced watertight closure of the dura mater after failure of the initial standard running suture technique. At least 4 times higher leak pressures were achieved by combining solid and fluid PRF as a simple onlay graft in less than 2 min. PRF can be harvested from 10 ml of donor blood after 8 min of centrifugation without any additional chemicals or pharmaceuticals needed. We will further investigate the potential of our combination of solid and fluid PRF as a highly effective, safe, simple, cost-effective substitute for dural reinforcement as well as augmentation of wound healing and for durable dural closure.

In our in vitro setting with a central defect of 20 mm, the maximum leak pressure observed after PRF-augmentation was 58 cmH20. Chauvet et al. in 2011, in a comparable in vitro testing environment, evaluated various commercial sealants and measured maximum leak pressures of 43 cmH20 [4] on a central linear dural-defect of 50 mm. Compared to other in vitro studies of artificial dura sealants, PRF in our experiments showed similar strengthening properties [23, 34].

Other studies have previously highlighted the importance of onlays to ensure a watertight dura closure in spinal [21, 25], supratentorial [29], skull base [28] and transsphenoidal surgery [26]. Intraoperatively, the sealants performance is associated with its adhesive capacity on the dura mater and elasticity, while postoperatively, a bio-integration of the sealant with the healing dura is crucial to preserve the water-tightness. Integrity of dura-healing is especially important in cases where reoperation in the same area may be necessary. Thus, an optimal sealant should not only offer good adhesive, mechanical and water-tightness properties but also promote and support the dura-healing process. In contrast to older protocols of platelet-rich plasma preparations, the second generation PRF, as initially described from Choukroun et al. in 2001, contains a higher number of leukocytes growth factors [24] and key immune cytokines and interconnects naturally with the surrounding tissues, promoting and accelerating all phases of wound healing [16]. PRF’s mechanical properties shown in our study, combined with its possible wound healing effect, makes it an interesting dura-sealing-onlay for further investigation.

A first-generation autologous fibrin tissue adhesive was used on cerebrospinal fluid leak in spinal cord, in a randomised controlled trial and proved safe and superior compared to commercial fibrin tissue adhesives [27]. In this study, 400 ml of patient’s whole blood was drawn prior to surgery, centrifuged and finally intermixed with calcium gluconate and human thrombin before its intraoperative use. The centrifugation procedure involved multiple steps including the removal of the patient’s leukocytes from the final material. In our study, we demonstrated the utilisation of a second-generation platelet concentrate without any use of chemical preservatives or anticoagulants which could be freshly prepared from the patient’s own blood during the operation. This process adds minimal additional costs, and the automated commercialised systems have simplified its utilization in the operating theatre.

In a recent study from Theys et al., solid PRF membranes were utilised as single onlays to reinforce the dural-closure in a clinical setting of 44 brain and spine operations. Among those, a CSF leak was present only after two endoscopic transsphenoidal pituitary and one spinal surgery. Adverse effects associated with the PRF augmentations were not observed. We propose that a combination of both PRF forms could offer a stronger sealing effect [33].

Our study results are limited due to the in vitro nature of our experiments. In vivo elaboration of the technique should be performed in the next step to validate the results for clinical use.

Another limitation of our experimental setup is the perfectly horizontal layer of dura mater which allowed an optimal application of the i-PRF. As i-PRF needs some time to solidify, clinically relevant applications on vertical or inclined dura orientations may present challenges and need further exploration. Our proposed method could be easily applied in spinal surgery where most cases are operated in a prone position. In supratentorial cranial surgery, transient intraoperative adjustments of the operating table could facilitate a proper application of i-PRF.

The lack of a standardised and widely accepted device for in vitro dura-closure testing in the literature, made us create a simple hydrostatic construct lacking electronic circuits, robust and reliable. Our design allowed a watertight fixation of the dura samples, preventing any lateral leakage that could affect our measurements.

In our future work, we aim to compare PRF augmentation with other commercial sealants, in the same experimental setting. If PRF proves to be at least similarly mechanically efficient, and additionally present better tissue-healing properties, it could become an alternative treating option of the neurosurgical armamentarium.

## Conclusions

We fabricated an experimental device that allowed us to stress-test our proposed PRF dura augmentation technique in a measurable fashion and compare it to standard dural closure. Autologous platelet rich fibrin augmentation (PRF), compared to the standard running suture technique, significantly reinforced the watertightness of the dura mater closure in vitro.

## Electronic supplementary material

ESM 2 (MPG 67.3 mb)
