# Correction to: Integrity of dural closure after autologous platelet rich fibrin augmentation: an in vitro study

**DOI:** 10.1007/s00701-021-04907-y

**Published:** 2021-09-03

**Authors:** I. Vasilikos, J. Beck, S. Ghanaati, J. Grauvogel, T. Nisyrios, K. Grapatsas, U. Hubbe

**Affiliations:** 1 Department of Neurosurgery, Medical Centre-University of Freiburg, Faculty of Medicine, University of Freiburg, Neurozentrum, Breisacher Str. 64, 79106 Freiburg, Germany; 2 Laboratory of Experimental Neurosurgery, Medical Centre-University of Freiburg, Faculty of Medicine, University of Freiburg, Neurozentrum, Breisacherstr. 64, Freiburg, Germany; 3 Frankfurt Oral Regenerative Medicine, Clinic for Maxillofacial and Plastic Surgery, Johann Wolfgang Goethe University, Frankfurt am Main, Germany; 4 Department of Oral and Craniomaxillofacial Surgery, University Medical Centre Freiburg, Freiburg, Germany; 5 Department of Thoracic Surgery, Faculty of Medicine, Medical Centre-University of Freiburg, Freiburg, Germany

**Correction to: Acta Neurochirurgica (2020) 162:737–743**

**https://doi.org/10.1007/s00701-020-04254-4**

The article “Integrity of dural closure after autologous platelet rich fibrin augmentation: an in vitro study”, written by Vasilikos, I., Beck, J., Ghanaati, S., Grauvogel, J., Nisyrios, T., Grapatsas, K., and Hubbe, U., was originally published Online First without Open Access. After publication in volume 162, issue 4, page 737–743 the author decided to opt for Open Choice and to make the article an Open Access publication. Therefore, the copyright of the article has been changed to © The Author(s) 2020 and the article is forthwith distributed under the terms of the Creative Commons Attribution 4.0 International License, which permits use, sharing, adaptation, distribution and reproduction in any medium or format, as long as you give appropriate credit to the original author(s) and the source, provide a link to the Creative Commons licence, and indicate if changes were made. The images or other third party material in this article are included in the article’s Creative Commons licence, unless indicated otherwise in a credit line to the material. If material is not included in the article’s Creative Commons licence and your intended use is not permitted by statutory regulation or exceeds the permitted use, you will need to obtain permission directly from the copyright holder. To view a copy of this licence, visit http://creativecommons.org/licenses/by/4.0. Open access funding enabled and organized by Projekt DEAL.

